# Evaluation of Pesticide Residues Occurrence in Random Samples of Organic Fruits and Vegetables Marketed in Poland

**DOI:** 10.3390/foods11131963

**Published:** 2022-07-01

**Authors:** Renata Kazimierczak, Dominika Średnicka-Tober, Jan Golba, Anna Nowacka, Agnieszka Hołodyńska-Kulas, Klaudia Kopczyńska, Rita Góralska-Walczak, Bogusław Gnusowski

**Affiliations:** 1 Department of Functional and Organic Food, Institute of Human Nutrition Sciences, Warsaw University of Life Sciences, Nowoursynowska 159c, 02-776 Warsaw, Poland; dominika_srednicka_tober@sggw.edu.pl (D.Ś.-T.); klaudia_kopczynska@sggw.edu.pl (K.K.); rita_goralska_walczak@sggw.edu.pl (R.G.-W.); 2 Organic Farming and Food Quality Department, Ministry of Agriculture and Rural Development, Wspólna 30, 00-930 Warsaw, Poland; jan.golba@gmail.com; 3 Institute of Plant Protection—National Research Institute, Władysława Węgorka 20, 60-318 Poznań, Poland; a.nowacka@iorpib.poznan.pl (A.N.); a.holodynska@iorpib.poznan.pl (A.H.-K.); b.gnusowski@op.pl (B.G.)

**Keywords:** carrot, beetroot, potato, apple, organic production, pesticide residues, LC-MS/MS, GC-ECD/NPD

## Abstract

In recent years, organic food, produced with the use of natural means and production methods, has been gaining more and more popularity among consumers. This is due, inter alia, to their belief that it is more abundant in health-promoting bioactive compounds and safer than conventional food. Consumers are increasingly aware of the harmfulness of plant protection products used in intensive agriculture, which are not allowed in organic production. At the same time, it is reported that a certain share of organic products on the EU market are contaminated with pesticide residues, which may raise consumer concerns and lead to a loss of trust in organic food. The aim of the present study was to investigate the problem of pesticide residues occurrence in random samples of organically produced fruits and vegetables (apples, potatoes, carrots, and beetroots) commonly used in the Polish households, and which are available directly from the organic producers in open markets in Poland. For simultaneous analysis of 375 pesticides, an LC-MS/MS system consisting of an Eksigent expert ultraLC 100-XL coupled to a triple quadrupole mass spectrometer QTRAP 6500 and GC Agilent 6890 N equipped with ECD/NPD system were used. Among the 96 vegetable and fruit samples studied, 89 samples (92.7%) were free from detectable pesticide residues, 7 samples (7.3%) of carrot (5) and potato (2) were contaminated, and in 1 of them (1.0%) the detected residues exceeded the maximum residue limit (MRL). None of the tested apple and beetroot samples were found to contain detectable residues. These findings are important for Polish consumers who look for high-quality organic food. However, the presence of detectable residues in a small proportion of the organic samples indicates a need to strengthen the monitoring of pesticides in organic crops, to educate farmers and to raise their awareness regarding the risks of unauthorized use of pesticides banned in organic farming, which can damage the reputation of the whole organic sector.

## 1. Introduction

Pesticides are agrochemicals widely used in crop cultivation, mainly to protect crops from pests and to control diseases and weeds. They often allow to produce higher yields while reducing labor input, which results in economic benefits for farmers. Using plant protection products is also broadly perceived as an important practice to meet plant health requirements, and to enable international trade of agricultural products. These are the main reasons for their widespread use in agriculture. Pesticides are also widely used outside the agricultural sector, for purposes such as the maintenance of green areas and sport fields, the maintenance of public buildings, weed abatement on roadsides and railroads, lawn maintenance, and wood or fabric preservation [1,2,3]. Unfortunately, the use of many pesticides poses a danger to the natural environment [4,5,6], and through inappropriate use, it leads to the presence of pesticide residues in crops and food products [7]. As a result of increasing scientific evidence on the harmfulness of pesticides, actions have been taken in the EU to reduce the risks associated with their use. EU policy priorities in this area are defined in Directive 2009/128/EC110. This document, among others, obliges member states to develop national action plans to reduce the risks associated with the use of pesticides and the impact of their use on human health and the environment, to promote the development and application of integrated pest management and alternative approaches or techniques to reduce dependence on the use of pesticides [8]. Currently, to increase the sustainability of the EU food supply chains, under the European Green Deal, the use of pesticides (mainly in the agricultural sector) is to be reduced by 50 percent by 2030 [9]. Through this action, a number of pesticides have already been abandoned in agricultural use. Altogether, among the 1378 active substances registered as potential agricultural pesticides, only 466 are approved for use in the EU. Over the last few years, some of the most dangerous pesticides have been replaced by more effective and safer alternatives, with faster biodegradation rates [6]. Despite this, Pesticide Action Network Europe (PAN) research shows that pesticides banned in the EU due to human health and environmental concerns are still often detected in foods available on the European market, since they are still broadly used in non-European countries exporting foods to the EU. According to the PAN study, residues of 74 banned pesticides were found in 6.2% of all tested samples in the EU, mainly in plant-based products (75.2%). In that, 50 and 48 banned pesticide residues were detected in fruits and vegetables, respectively, raising a question about the safety of these nutritionally important food groups, especially in light of the current recommendations for increasing their consumption in a healthy diet. It is of concern that toxic substances such as carbendazim, chlorate, and chlordecone are very often detected in foods available on the EU market. This also applies to other substances with proven toxicity for humans (e.g., DDT, hexachlorobenzene, anthraquinone, omethoate, and tricyclazole) and the environment (e.g., propargite and chlorfenapyr), even though these are less frequently detected [10].

Although the procedures for the approval of active substances of pesticides for agricultural use follow strict regulations, to ensure both their high effectiveness but also their environmental and consumer safety, serious concerns are continuously being raised around many pesticides regarding health risks related to the occupational exposure as well as the consumer exposure to their residues in food and drinking water [11,12]. Occurrence of pesticide residues in agricultural crops is often associated with the irrational use of pesticides, which can lead to both short- and long-term health and environmental effects [3,7,13,14]. Many of the effective crop pest and disease control agents have been classified as cancerogenic, toxic for reproduction, endocrine disruptors, and increasing the risk of immune and nervous system disorders and respiratory diseases [3,15,16,17,18,19,20]. The association between pesticide use and an increased risk of various cancers and other health problems has been supported by many studies [6,18,21,22,23]. A relatively new group of crop protection products, neonicotinoids, were shown to be toxic to birds and mammals, but also implicated in the decline of bees [15,24]. Pesticide exposure linked to multiple sources is especially dangerous for young children, making them vulnerable to potential neurological, neurodevelopmental, and many other effects [25,26,27]. Even modest exposure to pesticides early in life can impair a child’s brain and central nervous system development [28].

All of the above-described and other risks from exposure to pesticides give rise to concerns for consumers who demand food that does not contain agrochemical residues [29,30,31]. In the opinion of the majority of the consumers, organic food is safe and beneficial to their health, and they associate it with the absence of agricultural chemical residues, as well as with a natural taste and care for the environment. These perceived characteristics of organic food result from its restrictive production methods [32], which make it trusted by an increasing number of consumers worldwide [33].

At the same time, according to the available reports on the degree of food contamination with pesticides, a high and relatively constant percentage of food products with detectable pesticide residues can be found on the European market. This issue has been highlighted in recent years by the tests carried out annually in the framework of the mandatory control programs involving all EU and associated countries. This monitoring of the European market is the responsibility of the European Food Safety Authority (EFSA). The EFSA annual reports indicate that, in recent years, detectable residues of at least one pesticide have been found in 39.5–45.5% of the tested samples. Furthermore, that between 2.6 and 5.1% of the samples contained residues exceeding the legal limits (referred to as the MRL-maximum residue level in food of plant origin) (Table 1). Nonetheless, despite the fact that the use of synthetic pesticides in organic production is strictly prohibited according to EU Regulation 2018/848 [34], EFSA monitoring indicates that a certain share of organic samples contain detectable pesticide residues. The percentage of organic samples with detectable pesticide residues ranged between 8.3% in 2015 and 19.9% in 2020, and residues above the MRL were found in 0.7% in 2015 to 1.5% in 2020 of the tested organic samples.

It is worth noting that fruits and vegetables are among the food groups known to be most frequently contaminated with pesticide residues. This is supported by the meta-analysis of Barański et al., according to which 74.60% of conventional fruit samples contained pesticide residues, compared to 11.45% in the case of organic fruits. For vegetables it was 31.95% and 10.25% in the conventional and organic system, respectively [44]. Several reports also have confirmed that consumption of organic foods is associated with reduced consumer exposure to pesticide residues [25,45,46,47]. Even though the confirmed lower frequency of detection of pesticide residues in organic foods might be seen as positive information for consumers, due to the current legislation on organic production, it would seem logical that these residues should not occur at all in the organic products. Their occurrence can be caused by the spray drift from the conventional fields, environmental contamination or contamination during handling, packaging, storage, or processing of organic products, but can also result from the intentional use of unauthorized products or incorrect labelling of conventionally produced food as organic food [43].

Current dietary recommendations point to the importance of increasing fruit and vegetable consumption in a healthy diet [48]. According to the World Health Organization (WHO) and the Food and Agriculture Organization of the United Nations (FAO), the recommended fruit and vegetable consumption, excluding potatoes and other starchy tubers, is a minimum of 400 g (or five 80 g portions) per day [49]. As fruits and vegetables are the food groups most frequently contaminated with pesticide residues, the increase of fruit and vegetables consumption should be accompanied by their responsible choice (i.e., local, organic produce, significantly less frequently contaminated with pesticide residues) [50]. It is important to mention that organic fruits and vegetables also have been reported to contain, on average, more health-promoting bioactive substances (i.e., certain groups of phenolic compounds) [44,51,52], which is considered as one of the major factors responsible for their health-beneficial properties [44,53,54]. Consumers have the right to expect, when buying fruits and vegetables, that the products they get present a high nutritional and biological value and do not pose any risk to their health. To achieve this, food producers must comply with international and national regulations and standards to protect consumers and themselves from various hazards. This also applies to organic food producers, who should precisely follow the rules of the organic production methods in this regard. Care for food safety starts with production on the farm and extends throughout the whole supply chain [50].

In Poland, apples are among the fruits most valued by consumers, accounting for about 22.5% of total fresh fruit consumption per capita in households (not including bananas and citrus fruits) [55]. Their popularity in Poland results from the favorable soil and climatic conditions and thus a very strong and long tradition of both the production and the consumption of these fruits. Poland is one of the biggest apple producers in the EU and an important producer in the world [56]. Among the fresh vegetables, root vegetables, such as carrots and beetroots, and potatoes are consumed in great quantities by Polish consumers [55]. These products are also appreciated by consumers as well as farmers all over the globe.

The aim of the present study was to investigate the problem of pesticide residues occurrence in random samples of organically produced apples, potatoes, carrots, and beetroots, which are commonly used in Polish households and available directly from the organic producers in open markets in Poland. The outcomes on the safety status of these popular fruits and vegetables coming from organic production systems might be of great interest to consumers, who are increasingly searching for high-quality and pesticide-free organic foods.

## 2. Materials and Methods

### 2.1. Plant Material

Random samples (≥1 kg each) of fruits and vegetables (*n* = 96)—carrot roots (*Daucus carota*), beetroots (*Beta vulgaris* ssp. *vulgaris* var. *conditiva*), potato tubers (*Solanum tuberosum*), and apple fruits (*Malus domestica*)—were purchased directly from the organic producers selling their products in local open markets in Warsaw, Mazovia region (central Poland). All these fruits and vegetables were certified as organic by the Polish accredited organic certification bodies. Samples originated from the harvests of two subsequent years (2015–2016). After collection, samples were transported to the laboratory of the Institute of Plant Protection—National Research Institute (Department of Pesticide Residue Research) in Poznań, Poland, and after a very short storage at the controlled temperature of 4 °C (up to a maximum of 16 h after delivery of the samples to the laboratory), prepared for the analysis of the pesticide residues. In the first study year, samples from 8 batches of apples, 9 batches of carrots, and 11 batches of potatoes were collected, while in the second study year there were samples from 28 batches of beetroots, 22 batches of carrots, and 18 batches of potatoes (one sample was taken from each batch).

### 2.2. Reagents and Materials

HPLC for the UV gradient-grade acetonitrile was purchased from J.T. Baker (Phillipsburg, NJ, USA). Acetone for HPLC and ammonium formate, from Sigma-Aldrich, and LiChrosolv methanol hypergrade for LC-MS, Emplura n-hexane, and LiChropur formic acid for LC-MS were purchased from Merck (Darmstadt, Germany). Ultrapure water (18.2 MΩ cm) was obtained from a Milli-Q Academic A10 Water Purification System (Millipore Ltd., Bedford, MA, USA). QuEChERS reagents, SupelTM QuE Citrate Tube containing anhydrous magnesium sulphate (MgSO_4_), sodium chloride (NaCl), trisodium citrate dihydrate (NaCitrate tribasic dehydrate), disodium hydrogencitrate sesquihydrate (NaCitrate dibasic sesquihydrate), and clean-up mixture SupelTM QuE PSA Tube containing primary secondary amine sorbent (MgSO_4_ + PSA) were purchased from Supelco (Bellefonte, PA, USA). High-purity pesticide analytical standards (Dr. Ehrenstorfer, Augsburg, Germany) were purchased from LGC (Teddington, UK) and were stored at −20 °C in dark conditions.

### 2.3. Standard Solutions

The selection of the tested compounds was made on the basis of the list of active substances approved in the European Union, which is included in the Commission Implementing Regulation (EU) No 540/2011 [57], and also substances withdrawn from use or those currently used outside the European Union.

In total, 375 individual pesticide stock solutions with concentrations ranging from 46 to 2150 µg/mL were prepared in acetone, methanol, acetonitrile, or an acetone-methanol mixture, depending on the solubility of each compound. Stock solutions were stored at −20 °C in dark and dry conditions, in tightly closed glass containers, for 3–5 years, depending on the solvent used. From these solutions, standard mixtures consisting of 8 to 30 pesticides at concentrations of 20 or 40 µg/mL were made in acetone and used for instrumental analysis. The stability of the mixtures prepared from the stock solutions were checked by preparing new standard mixtures and comparing the detector responses for individual compounds. The differences between the means from five replicate measurements for each of the two solutions (old and new) did not exceed 10% (for analytes difficult to analyse—15%). The mix of 375 pesticides for the LC-MS/MS analysis was prepared from 18 stock mixtures combined into one at a concentration of 1000 ng/mL, which was diluted 2, 5, 10, 20, 100, and 200 times with acetone to obtain working solutions of 5, 10, 50, 100, 200, and 500 ng/mL. Calibration solutions were prepared on an ongoing basis by diluting 10 times the working solutions with the LC solvent mix consisting of Eluent A and Eluent B (19:1; *v*/*v*) (the composition of the eluents is described in the section on LC-MS/MS). Checking of the mixtures was performed periodically, every 3 months, comparing the newly prepared mixture with the previous one by making alternative injections of three concentrations corresponding to the range of the calibration curve (lowest, middle, and highest concentrations). Twelve mixes containing in total 205 pesticides were used for the GC-ECD/NPD analysis, and among them, 89 pesticides were only determined by this technique, while 116 pesticides were overlapped with LC-MS/MS. The working standard mixtures corresponding to 0.01, 0.02, 0.05, 0.1, 0.2, and 0.5 μg/mL were made by 40-, 100-, 200-, 400-, 1000-, and 2000-fold dilution of the standard mixes of 20 μg/mL, and by 80-, 200-, 400-, 800-, 2000-, and 4000-fold dilution of the standard mixes of 40 μg/mL with an acetone-n-hexane mixture (1:9, *v*/*v*). Isotopically labelled internal standards (IL-IS) were used for LC-MS/MS analysis. Atrazine D-5 (406 μg/mL) and linuron D-6 (398 μg/mL) stock solutions were prepared in acetone, while the carbendazim D-4 stock solution (338 μg/mL) in an acetone-methanol mixture (3:7, *v*/*v*). The IL-IS mix of 5 μg/mL was prepared in acetone and then diluted to 0.25 µg/mL with the same solvent.

### 2.4. Sample Preparation

For the sample preparation, the QuEChERS method using a citrate buffer was applied [58]. A 10.0 ± 0.1 g of a homogenized sample was weighed into a 50 mL disposable PTFE centrifuge tube and then 10 mL of acetonitrile and 100 µL of the isotopically labelled internal standard (IL-IS) mix (atrazine D5, carbendazim D4 and linuron D6) at a concentration of 5.0 µg/mL were added. The sample was shaken (HS 501 digital, IKA-Werke GmbH & CO. KG, Staufen, Germany) vigorously at 300 rpm for 5 min. Then a buffer-salt mixture (4 g MgSO_4_, 1 g NaCl, 1 g NaCitrate tribasic dehydrate and 0.5 g NaCitrate dibasic sesquihydrate) was added into the extraction tube and, after short mixing by hand, the sample was re-shaken as previously described. Afterwards, the sample was centrifuged (Rotina 420R, Hettich Zentrifugen, Tuttlingen, Germany) at 4500 rpm for 3.5 min at 15 ± 1 °C. Further analytical steps were different for the LC-MS/MS and GC-ECD/NPD technique and consisted of dilution or clean-up of the supernatant.

A 10-fold dilution prior the LC-MS/MS analysis was used to minimize the matrix effect. A 200 µL aliquot of the clear extract and 1800 µL of LC solvent mix (Eluent A + Eluent B; 19:1; *v*/*v*) were transferred into a 10 mL glass tube and filtered using a 0.2 µm × 13 mm syringe filter (Pall Co., Ann Arbor, MI, USA) into a 2 mL chromatography vial (Thermo Scientific, Langerwehe, Germany). The filtrate concentration was a 0.1 g sample per 1 mL solution. The d-SPE clean-up step was performed for the gas chromatography analysis. Then, 5 mL of centrifuged extract was transferred into a tube containing the clean-up mixture (900 mg MgSO_4_ with 150 mg PSA) and shaken at 2000 rpm for 1 min (Multi Reax, Heidolph, Schwabach, Germany). After re-centrifugation (in the way mentioned above), 1.5 mL of the extract was transferred to a 2 mL chromatography vial (Agilent, Santa Clara, CA, USA), acidified with 15 µL of a 5% formic acid solution in acetonitrile (10 µL per 1 mL of the extract) and evaporated to dryness under a gentle nitrogen stream. A dry residue was dissolved in 1.5 mL an acetone–n-hexane mixture (1:9, *v*/*v*). The final extract concentration was a 1 g sample per 1 mL solution.

### 2.5. Instrumental Analysis

#### 2.5.1. Liquid Chromatography with Tandem Mass Spectrometry (LC-MS/MS)

For simultaneous analysis of 286 pesticide residues, an LC-MS/MS system consisting of an Eksigent expert ultraLC 100-XL (Eksigent Technologies, Dublin, CA, USA) coupled to a triple quadrupole mass spectrometer QTRAP 6500 (AB Sciex, Foster City, CA, USA) was used. A 10-µL aliquot (cooled to 8 ± 1 °C in LC autosampler) was injected on a Kinetex core-shell C18 100 Å (100 mm × 2.1 mm, 2.6 µm) column (Phenomenex, Torrance, CA, USA) set up at 40 °C. The mobile phase gradient system was composed of Eluent A (a 0.1% solution of formic acid and 5 mM ammonium formate in water) and Eluent B (a 0.1% solution of formic acid and 5 mM ammonium formate in methanol) at a flow rate of 0.5 mL/min. The gradient elution was started with 95% A and 5% B and held for 1.0 min, then rising linearly to 10% A and 90% B in 21.0 min and held for 3.1 min. The composition of the mobile phase was returned to the starting condition over 1.9 min at a flow rate of 1 mL/min, followed by a column re-equilibration phase of 3.0 min. The total data acquisition time was 30.0 min. The LC gradient programme is shown in Table 2.

The triple quadrupole mass spectrometer was operated in positive electrospray ionization mode (ESI+), at a capillary voltage of 5000 V, desolvation temperature of 400 °C, entrance potential (EP) of 10 V, and with nitrogen as the curtain (CUR), nebulizer (GS1), and auxiliary (GS2) gas, at a pressure of 30, 60, and 50 psi, respectively. Nitrogen was also used as collision gas. The ionization and MS/MS collision energy settings were optimised while continuously infusing a pesticide solution at a flow rate of 10 µL/min via a syringe pump. The parent and two daughter ions were selected and the first one was used for quantification and the second one for confirmation. The precursor and product ions monitored, and the optimized declustering potential (DP), collision energy (CE), cell exit potential (CXP), and retention time (RT) are demonstrated for each pesticide in Table S1 in the Supplementary Material. Data acquisition was performed in the multiple reaction monitoring (MRM) mode. Analyst Software (version 1.6.2) and MultiQuant Software (version 3.0.2) from AB Sciex were used for data acquisition and processing, respectively.

#### 2.5.2. Gas Chromatography Coupled to Electron Capture Detection and Nitrogen-Phosphorus Detection (GC-ECD/NPD)

A GC Agilent 6890 N (Agilent Technologies, Palo Alto, CA, USA) equipped with a ^63^Ni ECD and NPD system operated at 300 °C and 320 °C was used for determination of pesticide residues. A 4-µL aliquot was injected using an HP 7683 Automatic Liquid Sampler (Agilent Technologies, Palo Alto, CA, USA) on a (5%-Phenyl)-methylpolysiloxane DB-5 (30 m × 0.53 mm × 0.88 µm) fused silica capillary column (Agilent J&W GC Column) connected to both detectors for simultaneous acquiring of chromatographic data. The pesticides were separated with a 60 min oven programming, starting with an initial temperature of 100 °C for 1 min with a ramp rate of 10 °C/min up to a temperature of 280 °C with a hold time of 41 min. The injector was operated in splitless mode at 250 °C. Helium (99.999%) with a flow rate of 3.8 mL/min was used as the carrier gas. For the ECD, nitrogen (99.999%), as a make-up gas, flow was 60.0 mL/min. For the NPD, nitrogen as auxiliary gas (99.999%), hydrogen (99.999%), and air (99.995%) flows were 10.0, 3.0, and 120.0 mL/min, respectively. The analytical results were confirmed by the previous double measurement of the detector response for one compound; for example, for chlorpyrifos (Table S2 in the Supplementary Material), two results were obtained—on the ECD and NPD. If the results obtained in this way were within the tolerable error (y = 12.5x − 0.30103 where x is average result) and the retention times for the compound showed agreement (±0.1 min), this was taken as a confirmation of the result. In case a compound was tested on only one detector, the analytical result was confirmed by re-running samples on a different polarity column using the identical chromatographic parameters (same acceptance criteria as above). The second Agilent 6890 N system was equipped with a 35%-diphenyl/65%-dimethylpolysiloxan DB-35 (30 m × 0.32 mm × 0.5 µm) fused silica capillary column (Agilent J&W GC Column). GC ChemStation Rev. B. 04.03-SP2 (108) software for the instrument control and data acquisition and evaluation was used. The system suitability test mix, containing chlorothalonil, chlorpyrifos, chlorpyrifos methyl, pp-DDD, o,p′-DDT, p,p-DDT, dimethoathe, lindan, phosalone, and propoxur, was injected at the beginning and the end of each chromatographic batch to check the performance of the GC-ECD/NPD system. The retention times (RT) and detector type for analysis of each pesticide by GC-ECD/NPD are shown in Table S2 in the Supplementary Material.

### 2.6. Method Validation Procedure and Real Sample Analysis

The method was validated for 375 analysed pesticide residues with representative commodities from the commodity group of high-water content (tomatoes, potatoes) at the levels of 0.01 mg/kg, 0.02 mg/kg, or 0.20 mg/kg for the GC-ECD/NPD technique and 0.01 and 0.05 mg/kg for the LC-MS/MS technique according to the SANCO/12571/2013 document [59]. The method validation parameters (sensitivity/linearity, specificity, trueness/average recovery, precision, LOQ, matrix effect, and robustness) met the criteria for the tested compounds specified in the document. For all pesticide residues, the limit of quantitation (LOQ) was set at a concentration of 0.01 mg/kg. The recoveries were in the range of 70–120% and precision (RSD) did not exceed 20%. Linearity was characterised by R^2^ ≥ 0.995. The matrix effect was evaluated through comparison of the slopes of the standards in a solvent with matrix-matched standards. The suitability of the method for testing of the pesticide residues was confirmed, in the years 2014–2016, in three rounds of proficiency tests (EUPT-FC-16, EUPT-FV17, and EUPT-FV18) organised by the European Union Reference Laboratory for fruits and vegetables. Pesticide residues in the analysed samples were determined by LC-MS/MS and GC-ECD/NPD. Each analytical batch was comprised of calibration mixes, study and fortification samples, and blanks, to eliminate the possibility of analytes carry-over. A multi-level calibration was used to calculate the results. The calibration curve solutions in pure solvent for LC-MS/MS were prepared at the day of the analysis, in 2 mL chromatography vials, from the working solution in acetone, by adding 100 µL of the standard mixture (ranging from 5 to 500 ng/mL), 880 µL of the LC solvent mix (Eluent A + Eluent B; 19:1; *v*/*v*), and 20 µL of the 0.25 µg/mL IL-IS mix. For the LC-MS/MS system, 6-point calibration, with the standard mix consisting of 286 compounds at concentrations of 0.5, 1, 5, 10, 20, and 50 ng/mL, corresponding to 0.005, 0.01, 0.05, 0.10, 0.20, and 0.50 mg pesticide per kg sample, was used. The IL-IS mix was applied for quality control of the whole analytical process. The GC-ECD/NPD system was calibrated with many mixtures. The main pesticide mix, diluted to 0.01, 0.02, 0.05, 0.1, 0.2, and 0.5 µg/mL, corresponding to 0.01, 0.02, 0.05, 0.1, 0.2, and 0.5 mg pesticide per kg sample, was used for the 6-point calibration. The other pesticide mixes at the concentration of 0.01 and 0.05 µg/mL, corresponding to 0.01 and 0.05 mg pesticide per kg sample, were applied for the 2-point calibration.

**Figure 1:**
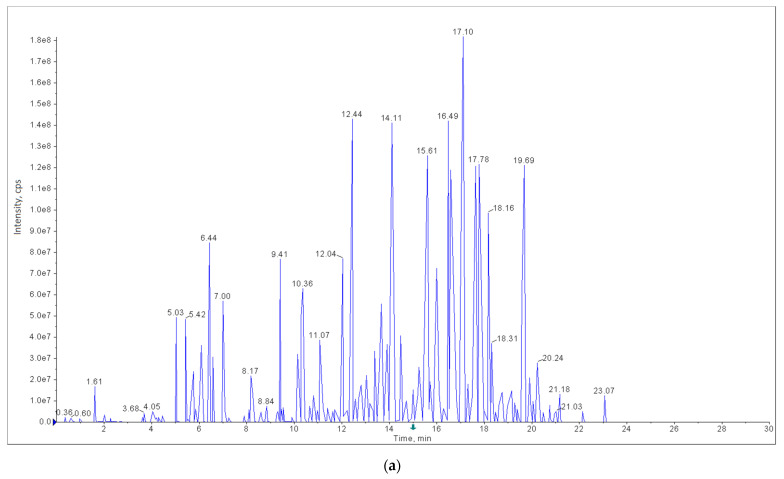

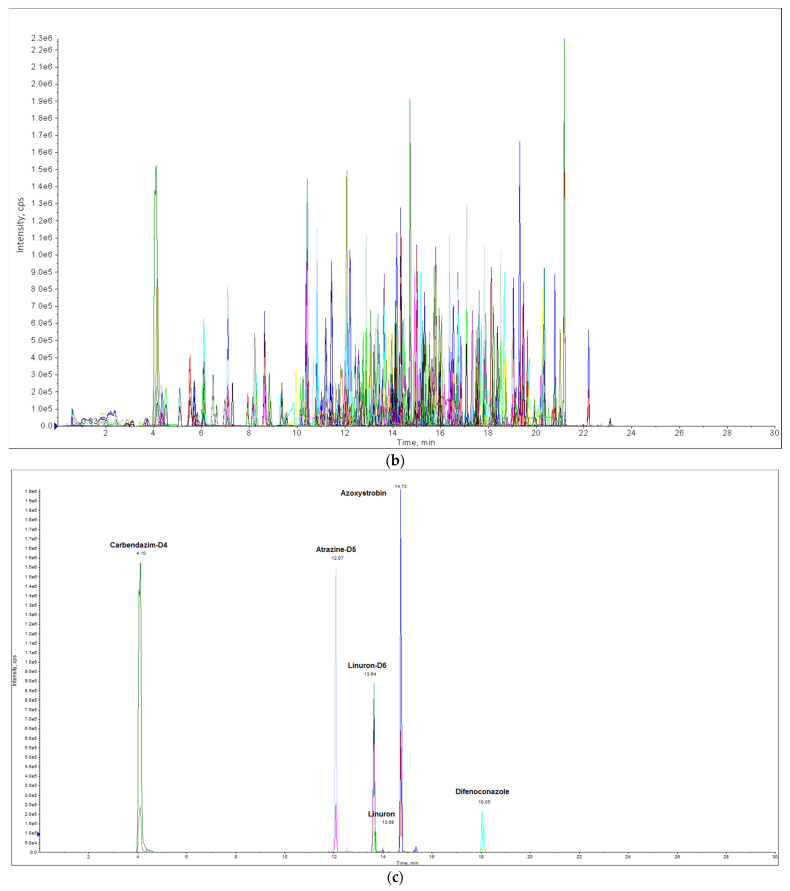

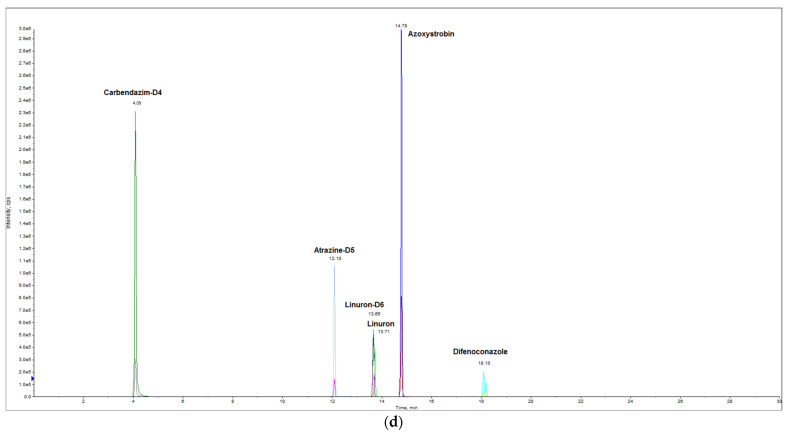
Multiple reaction monitoring (MRM) chromatograms (Analyst software): (**a**) total ion current chromatogram of the standard calibration mix (1 ng/mL); (**b**) extracted ion chromatogram of the standard calibration mix (1 ng/mL); (**c**) extracted ion chromatogram of azoxystrobin, difeconazole, linuron (1 ng/mL), and isotopically labelled internal standards; (**d**) extracted ion chromatogram of azoxystrobin (0.019 mg/kg), difeconazole (0.010 mg/kg), linuron (0.027 mg/kg), and isotopically labelled internal standards in one of the analysed carrot samples.

**Figure 2:**
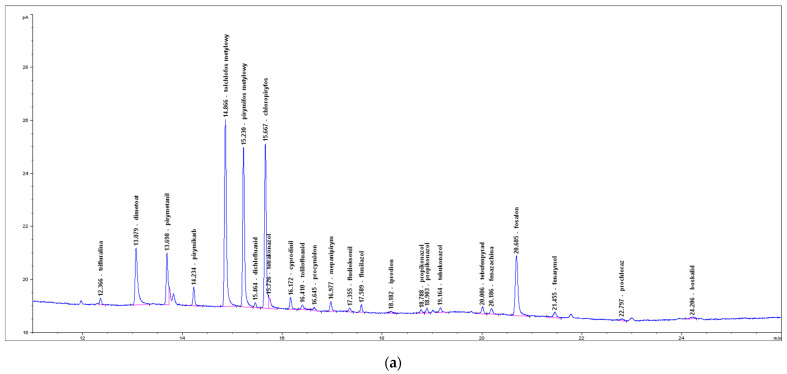

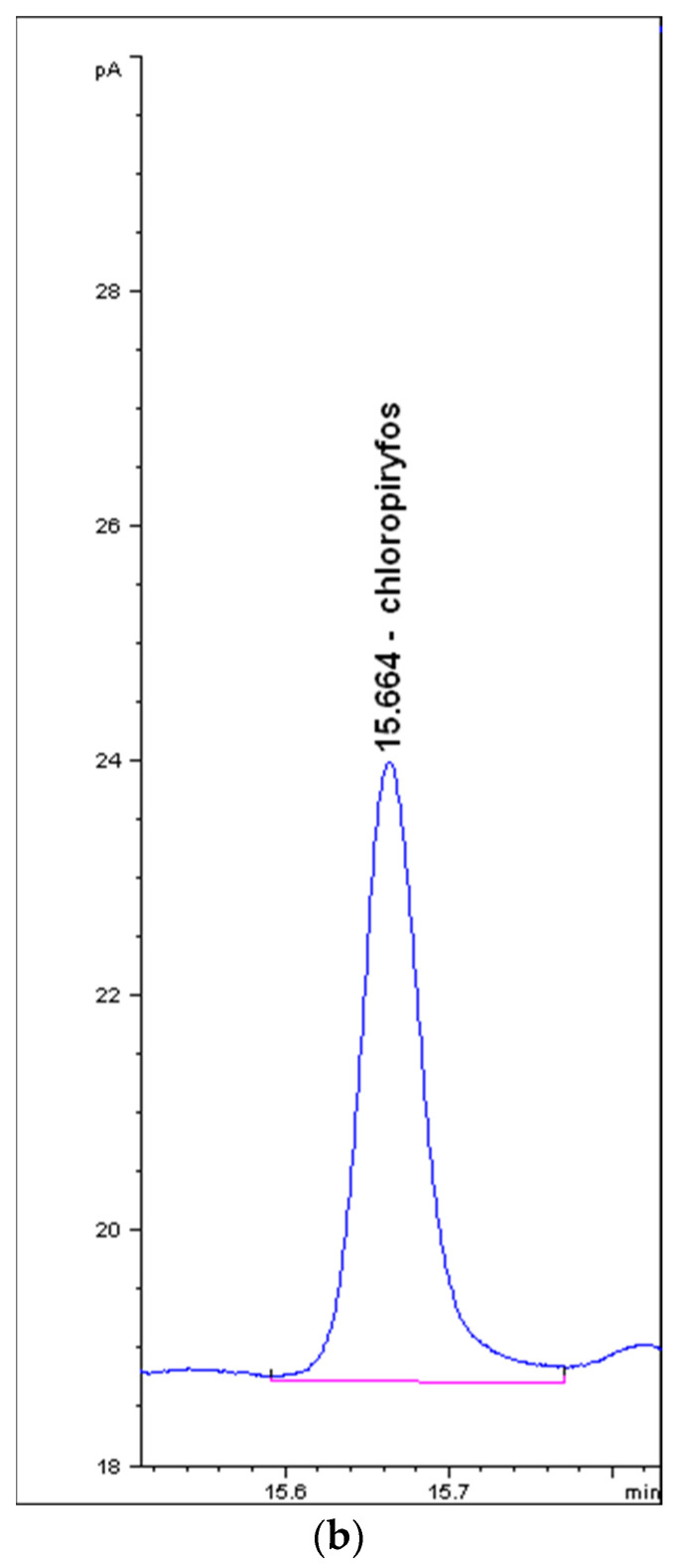
GC-NPD chromatogram of the selected pesticides: (**a**) the standard mix at 0.01 mg/kg; (**b**) chlorpyrifos residue at 0.010 mg/kg (the carrot sample).

**Figure 3:**
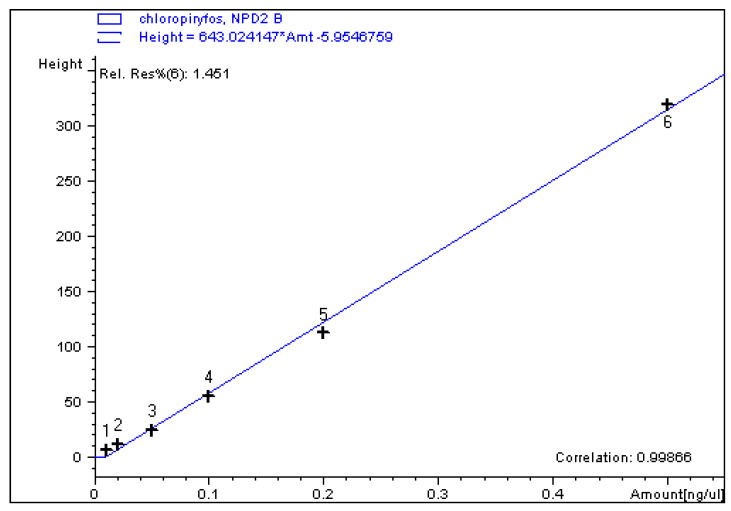
The 6-point calibration curve with the R^2^ for chlorpyrifos on GC-NPD.

**Figure 4:**
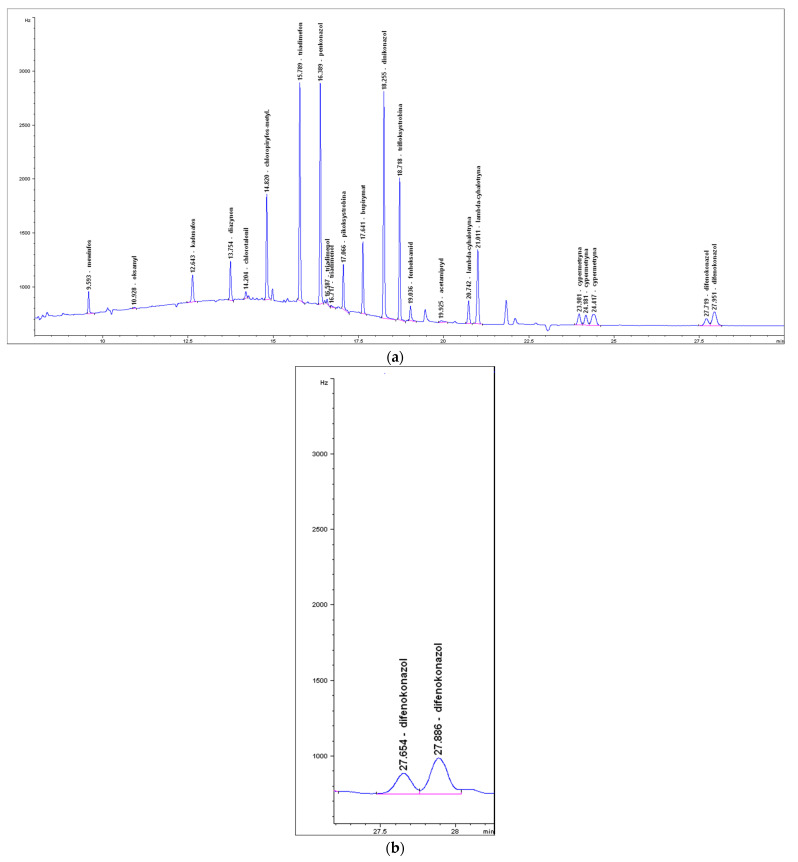
GC-ECD chromatogram of the selected pesticides: (**a**) the standard mix at 0.01 mg/kg; (**b**) difenoconazole residue at 0.010 mg/kg (the carrot sample).

Exemplary calibration curves, chromatograms of the standard calibration mixtures, and the carrot samples in which the pesticide residues were detected, are shown in Figure 1, Figures S1–S3 (Supplementary Material) (LC-MS/MS) and in Figure 2, Figure 3 and Figure 4 (GC-ECD/NPD).

## 3. Results and Discussion

As reported in the scientific literature, the presence of pesticide residues in fruit and vegetables is considered as one of the major food safety concerns nowadays. There is increasing evidence that the choice of organically produced foods may help to reduce consumers’ pesticide exposure [25,46,47,60]. This is confirmed by EFSA’s annual monitoring of the occurrence of pesticide residues in food in the EU. According to the EFSA reports from 2015 and 2016, among over 84,000 samples tested each year, 43.9 and 45.5%, respectively, overall, and 8.3 and 15.6% of organic samples, contained quantified pesticide residues. Residues exceeding the MRLs were found in 2.8% (2015) and 3.8% (2016) of the samples. In samples from organic production, it was 0.7% and 1.3%, respectively, in these years [38,39]. The most recent EFSA report shows that 40.3% of all tested samples were contaminated by detectable pesticide residues, and in the case of organic samples, it was 19.9%. In these, residues in 5.1% of the samples, including 1.5% of samples from organic production, exceeded the MRLs [43].

In our study, the collected organic carrot, potato, beetroot, and apple samples were tested for 375 active substances of synthetic crop protection agents, including herbicides, insecticides, and fungicides widely used in conventional agriculture, but prohibited in organic production. The method validation parameters (sensitivity/linearity, specificity, trueness/average recovery, precision, LOQ, matrix effect, and robustness) met the criteria for the tested compounds specified in the document SANCO/12571/2013 [59]. Out of the 96 organic fruit and vegetable samples analyzed, 89 samples (92.7%) were free from detectable pesticide residues, 7 samples (7.3%) were contaminated (5 carrot and 2 potato samples), and in 1 of them (1.0%) (carrot sample) the detected residues exceeded the maximum residue limit (MRL) (Table 3 and Table 4).

The noticeable difference between the data published by the European Food Safety Authority (EFSA) and our findings on the percentage of organic samples contaminated with pesticide residues might suggest that the Polish samples are contaminated less frequently than the average European samples. However, it should be taken into consideration that (a) the present study included only a few vegetable and fruit species (and not necessarily those representing the greatest problem of contamination with pesticides); and (b) the organic samples in the present study came exclusively from the local farmers’ market in Warsaw and thus may not be representative of the overall organic food market, including all other food sources. Interestingly, the share of foods from Poland in the overall European monitoring is rather limited. According to the most recent EFSA report, only 3246 samples (both organic and conventional) from Poland were included in the analyses in 2020, while, for example, from Germany, Italy, and France, it was 18,837, 8360, and 7830, respectively, and 9370 samples were analyzed in the case of Bulgaria, which is a smaller country compared to Poland. Per 100,000 inhabitants in Poland, this represented only 8.55 samples, while per 100,000 inhabitants in Germany it was 22.65, and in Bulgaria over 134 samples [43].

It is very important to note that the MRLs have been established for the foods coming from conventional production. In the case of an organic system, it is expected that products would be free from residues, which in practice means that the residues would not exceed the limit of quantification of the method. In many countries the level of up to 0.01 mg/kg f.w. is an accepted value, considered as a proof that no banned pesticides have been used in the production of the tested organic product. The same limits are commonly applied for the foods for infants [61] and for the products for which no MRLs have been set [62]. Looking at the results obtained in this study, we can observe that in the case of 6 out of 7 samples with detected pesticide residues, the 0.01 mg/kg f.w. residue level was exceeded (Table 3 and Table 4).

Despite the avoidance of pesticides in organic production, agricultural practices do not guarantee products completely free from residues. This is, among others, due to the fact that organic farms are often located within the agroecosystems where pesticides and other chemicals are widely used [63]. However, contamination with pesticides not permitted in organic agricultural production should result in the foods losing their organic status [64].

The results obtained in our study are consistent with the findings summarized in two large meta-analyses on various measures of the quality and safety of organic vs. non-organically produced foods. According to Smith-Spangler et al. [46], detectable pesticide residues were found in 7% of organically produced samples. In the meta-analysis of Barański et al. [44], it was shown that the incidence of pesticide residue detection in organic samples reached 10.55%. According to the same study, the frequency of contamination of the conventional samples reached on average 46.35%, and from that 31.95% in vegetables and nearly 75% in fruits.

The results were calculated comparing to calibration curve solutions in pure solvent for both LC-MS/MS and GC-ECD/NPD (see Section 2.6), since the matrix effect tested during the validation did not exceed 20% for the compounds detected. In our study, 5 samples of carrots out of 31 tested and 2 samples of potatoes out of 29 tested were identified as containing detectable pesticide residues. At the same time, none of the 8 samples of apples and 28 samples of beetroot were found to contain residues of these agrochemicals (Table 3 and Table 4). The most frequently detected pesticide was the organophosphate insecticide chlorpyrifos. Residues of this agent were present in 4 samples: in 1 sample of potato from 2015 (Table 3) and 3 samples of carrots from 2016 (Table 4). Two samples of carrot contained the systemic fungicide azoxystrobin and at the same time the broad-spectrum fungicide used for disease control in field crops—difenoconazole. In both cases, these compounds were detected in carrots grown in 2016 (Table 4). Another contaminated carrot sample also came from 2016 and contained residues of a triazole fungicide—propiconazole—and a selective herbicide—linuron. Among all the tested potato samples, one sample from 2015 contained propamocarb (Table 3) and one sample from 2016 contained imidacloprid (Table 4).

It is worth pointing that some of the pesticides detected in the carrot and potato samples are no longer on the European market. Linuron was withdrawn from sale in the EU in June 2017, as a substance classified as an endocrine disruptor and a carcinogen, toxic for reproduction, and posing high risk to birds, wild mammals, non-target arthropods, and soil microorganisms [65]. In 2019, a popular fungicide propiconazole was also withdrawn from the EU market due to its potential for reproductive toxicity, and due to the identified problem of groundwater contamination by propiconazole metabolites [8,66]. Another pesticide, chlorpyrifos, detected in the highest number of samples in our study (with residue level exceeding MRL in one carrot sample), was withdrawn from the EU market in 2020, due to its genotoxic potential, reproductive toxicity, and the identified association between exposure to this pesticide and adverse neurodevelopmental outcomes in children [67].

Even though none of the pesticides detected in this study was banned in the EU at the time when the plant material for the study was collected (2015–2016), it should be pointed out that none of the detected substances was allowed in cultivation under the EU regulation on organic production [32].

## 4. Conclusions

It can be concluded that the vast majority of organic fruits (apples), vegetables (carrot, beetroot), and potatoes purchased directly from Polish organic farmers do not contain detectable pesticide residues. This finding is important both for Polish consumers and retailers who look for high-quality organic foods. However, it appears that a small proportion of the organic samples tested in this study contained detectable residues of chemicals not authorized for use in organic production. Regardless of the reasons (i.e., deliberate use of unauthorized products, limited knowledge of farmers, spray drift from the conventional neighboring fields), this may raise concerns among consumers and may seriously threaten their trust in organic food quality and safety. Therefore, there is a need to strengthen the monitoring of pesticides in organic crops, to educate farmers, and to raise their awareness regarding the risks of unauthorized use of pesticides banned in organic farming, which can damage the reputation of the whole organic sector.

## Figures and Tables

**Table 1:** EFSA monitoring data on residues of pesticides in food samples 2012–2020.

Year	Number of Samples Tested	Samples with Pesticide Residues below the MRL ^1^	Samples with Pesticide Residues Exceeding the MRL	Number of Organic Samples Tested (% of Total Tested)	Organic Samples with Pesticide Residues below the MRL	Organic Samples with Pesticide Residues Exceeding the MRL	References
2012	78,390	43.8%	3.1%	4576 (5.8%)	14.1%	0.8%	[35]
2013	80,967	42.8%	2.6%	4620 (5.7%)	15.5%	0.8%	[36]
2014	82,649	43.4%	2.9%	4792 (5.8%)	12.4%	1.2%	[37]
2015	84,341	43.9%	2.8%	5331 (6.4%)	8.3%	0.7%	[38]
2016	84,657	45.5%	3.8%	5495 (6.5%)	15.6%	1.3%	[39]
2017	88,247	41.8%	4.1%	5806 (6.6%)	13.7%	1.5%	[40]
2018	91,015	43.3%	4.5%	5735 (6.3%)	15.2%	1.4%	[41]
2019	96,302	39.5%	3.9%	6048 (6.2%)	13.1%	1.3%	[42]
2020	88,141	40.3%	5.1%	5783 (6.5%)	19.9%	1.5%	[43]

^1^ MRL—the ‘maximum residue level’ is defined as the upper legal level of concentration for a pesticide residue in or on food or feed set in accordance with Regulation (EC) No. 396/2005, based on good agricultural practice and the lowest consumer exposure necessary to protect vulnerable consumers [43].

**Table 2:** LC gradient programme.

Time (min)	Flow (mL/min)	Eluent A (%)	Eluent B (%)
0.00	0.50	95	5
1.00	0.50	95	5
22.00	0.50	10	90
25.00	0.50	10	90
25.10	1.00	95	5
27.00	0.50	95	5
30.00	0.50	95	5

**Table 3:** Pesticide residues in the samples of apple fruits, potato tubers, and carrot roots from an organic local open market in Warsaw, Poland (2015).

Plant Material	Number of Samples Tested	Number of Samples with Detected Pesticide Residues ^1^	Compounds Detected and Level of Contamination (mg/kg f.w. ^2^)	MRL for CONV ^3^ (mg/kg f.w.)	Samples with Pesticide Residues Exceeding the MRL for CONV	MRL for ORG ^4^ (mg/kg f.w.)	Samples with Pesticide Residues Exceeding the MRL for ORG
Apple fruit	8	-	-	-	-	-	-
Potato tuber	11	1	propamokarb (0.018)	0.30	-	0.01	+
Carrot root	9	1	chlorpyrifos (0.037)	0.10	-	0.01	+

^1^ Samples tested for the presence of 375 active substances, LOQ 0.01 mg/kg f.w.; ^2^ f.w.—fresh weight; ^3^ Maximum residue level established for conventional (CONV) foods; ^4^ Maximum residue level used for organic (ORG) products.

**Table 4:** Pesticide residues in the samples of apple fruits, carrot roots, and beetroots from an organic local open market in Warsaw, Poland (2016).

Plant Material	Number of Samples Tested	Number of Samples with Detected Pesticide Residues ^1^	Compounds Detectedand Level of Contamination (mg/kg f.w. ^2^)	MRL for CONV ^3^ (mg/kg f.w.)	Samples with Pesticide Residues Exceeding the MRL for CONV	MRL for ORG ^4^ (mg/kg f.w.)	Samples with Pesticide Residues Exceeding the MRL for ORG
Potato tuber	18	1	imidacloprid (0.010)	0.50	-	0.01	-
Beetroot	28	-	-	-	-		-
Carrot root	22	4	Smpl 1: azoxystrobin (0.017)	1.00	-	0.01	+
			Smpl 1: difenoconazole (0.040)	0.40	-	0.01	+
			Smpl 1: chlorpyrifos (0.260)	0.10	+	0.01	+
			Smpl 2: azoxystrobin (0.019)	1.00	-	0.01	+
			Smpl 2: difenoconazole (0.010)	0.40	-	0.01	-
			Smpl 2: linuron (0.027)	0.20	-	0.01	+
			Smpl 3: chlorpyrifos (0.010)	0.10	-	0.01	-
			Smpl 4: propiconazole (0.031)	0.05	-	0.01	+
			Smpl 4: chlorpyrifos (0.034)	0.10	-	0.01	+

^1^ Samples tested for the presence of 375 active substances, LOQ 0.01 mg/kg f.w.; ^2^ f.w.—fresh weight; ^3^ Maximum residue level established for conventional (CONV) foods; ^4^ Maximum residue level used for organic (ORG) products.

## Data Availability

Data will be made available upon reasonable request by the corresponding author (Renata Kazimierczak).

## Supplementary Materials

The following supporting information can be downloaded at: https://www.mdpi.com/article/10.3390/foods11131963/s1, Table S1: Multiple reaction monitoring (MRM) conditions of each target pesticide for LC-MS/MS analysis; Table S2: Retention times (RT) and detector type of each pesticide analysed by GC-ECD/NPD; Figure S1: Calibration curve and extracted ion chromatograms of azoxystrobin (MultiQuant software): (a) Calibration curve (0.5–50 ng/mL); (b) Extracted ion chromatogram at 0.5 ng/mL; (c) Extracted ion chromatogram at 5 ng/mL; (d) Extracted ion chromatogram at 50 ng/mL; (e) Extracted ion chromatogram of azoxystrobin residues at 0.019 mg/kg (the carrot sample); Figure S2: Calibration curve and extracted ion chromatograms of difenoconazole (MultiQuant software): (a) Calibration curve (0.5–50 ng/mL); (b) Extracted ion chromatogram at 0.5 ng/mL; (c) Extracted ion chromatogram at 5 ng/mL; (d) Extracted ion chromatogram at 50 ng/mL; (e) Extracted ion chromatogram of difenoconazole residues at 0.010 mg/kg (the carrot sample); Figure S3: Calibration curve and extracted ion chromatograms of linuron (MultiQuant software): (a) Calibration curve (0.5–50 ng/mL); (b) Extracted ion chromatogram at 0.5 ng/mL; (c) Extracted ion chromatogram at 5 ng/mL; (d) Extracted ion chromatogram at 50 ng/mL; (e) Extracted ion chromatogram of linuron residues at 0.027 mg/kg (the carrot sample).
