# Supplementary material for: Evaluation of Pesticide Residues Occurrence in Random Samples of Organic Fruits and Vegetables Marketed in Poland
Source: Foods. 2022 Jul 1;11(13):1963. doi: 10.3390/foods11131963 (PMC9265439; doi:10.3390/foods11131963)
Supplement: Supplementary file 1 [file foods-11-01963-s001.zip › foods-1772372-supplementary.pdf]

foods

Supplementary Materials

Evaluation of Pesticide Residues Occurrence in Random Samples of Organic Fruits and Vegetables Marketed in Poland

Renata Kazimierczak [1,*], Dominika Średnicka-Tober [1], Jan Golba [2], Anna Nowacka [3], Agnieszka Hołodyńska-Kulas [3], Klaudia Kopczyńska [1], Rita Góralska-Walczak [1] and Bogusław Gnusowski [3]

[1] Department of Functional and Organic Food, Institute of Human Nutrition Sciences, Warsaw University of Life Sciences, Nowoursynowska 159c, 02-776 Warsaw, Poland; dominika_srednicka_tober@sggw.edu.pl (D.Ś.-T.); klaudia_kopczynska@sggw.edu.pl (K.K.); rita_goralska_walczak@sggw.edu.pl (R.G.-W.)

[2] Organic Farming and Food Quality Department, Ministry of Agriculture and Rural Development, Wspólna 30, 00-930 Warsaw, Poland; jan.golba@gmail.com

[3] Institute of Plant Protection—National Research Institute, Władysława Węgorka 20, 60-318 Poznań, Poland; a.nowacka@iorpib.poznan.pl (A.N.); a.holodynska@iorpib.poznan.pl (A.H.-K.); b.gnusowski@op.pl (B.G.)

* Correspondence: renata_kazimierczak@sggw.edu.pl; Tel.: +48-22-593-7035

Table S1. Multiple reaction monitoring (MRM) conditions of each target pesticide for LC-MS/MS analysis.

**Mass Spectrometer**	**AB Sciex QTRAP 6500 LC-MS/MS system**					
**Ionization**	**Positive Electrospray Ionization, ESI+ (Multiple Reaction Monitoring Mode)**					
**Target compound**	**Quantification**	**Qualification**	RT[1] (min)	DP[2] (V)	CE[3] (V)	CXP[4] (V)
3-Hydroxycarbofuran	238.1>181.1	238.1>163.0	6.96	46/46	17/21	10/10
Acephate	184.0>143.0	184.0>95.0	1.20	26/26	13/33	8/14
Acetamiprid	223.0>125.9	223.0>99.0	7.06	80/80	27/51	6/5
Acibenzolar-S-methyl	211.0>136.0	211.0>91.0	13.53	96/96	39/28	8/11
Aldicarb	208.1>116.1	208.1>89.0	8.21	26/26	11/21	8/14
Aldicarb-sulfone	223.1>148.0	223.1>86.0	3.08	36/36	13/23	8/14
Aldicarb-sulfoxide	207.0>132.0	207.0>88.9	2.62	36/36	11/21	8/10
Ametryn	228.1>186.0	228.1>96.0	12.22	86/86	25/33	10/14
Amidosulfuron	370.0>261.0	370.0>218.0	11.46	25/25	19/33	14/12
Aminocarb	209.1>137.1	209.1>152.1	2.23	56/56	33/19	8/10
Atrazine	216.1>174.0	216.1>104.0	12.16	51/51	23/37	10/12
Azaconazole	300.0>159.0	300.0>231.0	12.89	86/86	37/23	10/12
Azoxystrobin	404.1>371.9	404.1>344.0	14.77	61/61	19/33	20/18
Benalaxyl	326.1>148.1	326.1>208.2	17.13	76/76	27/21	8/12
Bendiocarb	224.1>167.1	224.1>109.0	10.35	36/36	13/23	10/8
Bensulfuron-methyl	411.0>149.0	411.0>118.9	14.02	101/101	27/57	8/12
Benthiavalicarb-isopropyl	382.1>180.0	382.1>116.0	15.30	91/91	41/27	10/6
Benzoximate	364.0>199.0	364.0>104.9	17.85	56/56	13/35	10/12
Bifenazate	301.1>170.0	301.1>151.9	15.66	76/76	27/55	10/8
Bitertanol	338.0>269.0	338.0>70.0	17.71	50/50	15/25	16/16
Boscalid	343.0>307.0	343.0>140.0	14.72	116/116	27/25	16/8
Bromacil	261.0>204.9	261.0>187.9	9.96	36/36	19/37	12/10
Bromuconazole	378.0>159.0	378.0>70.0	15.14/16.03	91/91	35/61	10/8
Bupirimate	317.1>166.1	317.1>108.1	15.54	91/91	31/33	10/8
Buprofezin	306.1>201.1	306.1>116.1	18.70	51/51	17/21	10/6
Butafenacil	492.1>331.0	492.1>180.0	16.03	46/46	33/57	18/10
Butoxycarboxim	223.1>106.0	223.1>166.1	2.89	40/40	11/15	6/8
Buturon	237.0>84.0	237.0>125.9	12.55	91/91	21/39	14/6
Cadusafos	271.0>158.9	271.0>130.9	17.93	36/36	19/31	10/6
Carbaryl	202.1>145.1	202.1>127.1	11.08	21/21	15/39	8/8
Carbendazim	192.0>160.1	192.0>132.1	4.13	71/71	27/43	10/8
Carbetamide	237.1>192.1	237.1>118.1	9.36	36/36	13/17	12/6
Carbofuran	222.1>165.1	222.1>123	10.42	16/16	17/29	10/6
Carboxine	236.1>143.0	236.1>87.0	10.85	16/16	21/31	10/10
Carfentrazone-ethyl	412.0>345.9	412.0>365.9	16.77	126/126	33/25	18/20
Chlorbromuron	292.9>204.0	292.9>182.0	14.14	51/51	28/25	10/9
Chloridazon	222.0>104.0	222.0>92.0	6.61	11/11	31/33	12/14
Chloroanthraniliprol	483.9>452.9	483.9>285.8	13.85	96/96	23/19	24/16

Chloroxuron	291.0>72.0	291.0>218.0	15.37	106/106	23/33	8/11
Chlorsulfuron	358.0>167.0	358.0>141.1	11.85	25/25	23/23	10/6
Chlortoluron	213.1>72.0	213.1>46.0	11.64	76/76	21/37	10/12
Chromafenozide	395.2>175.0	395.2>339.1	15.99	40/40	21/11	10/19
Cinosulfuron	414.0>183.0	414.0>215.0	10.69	76/76	23/22	10/11
Clodinafop-propargyl	350.0>266.0	350.0>90.9	16.80	101/101	21/35	14/14
Clofentezine	303.0>138.0	303.0>101.9	17.53	51/51	19/49	8/12
Clomazone	240.0>125.0	240.0>89.0	13.41	66/66	27/65	6/10
Clothianidin	250.0>169.0	250.0>132.0	5.80	6/6	19/21	10/6
Crimidin	172.0>106.9	172.0>95.1	6.98	91/91	35/31	14/8
Cyazofamid	325.1>108.0	325.1>261.0	16.04	16/16	19/13	8/14
Cycloxydim	326.2>280.1	326.2>180.0	18.23	91/91	19/27	14/10
Cycluron	199.1>89.0	199.1>72.0	12.62	71/71	21/29	10/10
Cyflufenamid	413.1>241.0	413.1>203.0	17.93	66/66	31/55	14/12
Cymoxanil	199.0>128.0	199.0>111.0	7.07	81/81	13/25	4/4
Cyprazine	228.1>186.0	228.1>108.0	12.23	96/96	25/33	10/12
Cyproconazole	292.0>70.0	292.0>125.0	14.89/15.03	61/61	23/45	8/6
Cyprodinil	226.1>93.0	226.1>77.0	15.26	71/71	43/61	12/12
Demeton-S	259.0>88.9	259.0>61.0	13.61	21/21	21/49	14/10
Demeton-S-methyl-sulfone	263.0>169.0	263.0>108.9	4.50	76/76	21/37	12/14
Desmedipham	318.1>182.1	318.1>136.0	13.66	16/16	19/37	10/6
Dichlofluanid	350.0>223.9	350.0>123.0	15.67	60/60	21/39	12/6
Diclobutrazol	328.0>69.9	328.0>159.0	16.65	85/85	58/48	8/8
Dicrotophos	238.0>112.1	238.0>193.0	6.06	46/46	17/13	8/10
Diethofencarb	268.1>226.1	268.1>180.0	14.16	26/26	13/25	12/10
Difenoconazole	406.0>251.0	406.0>188.0	18.11	96/96	35/59	14/10
Difenoxuron	287.0>123.0	287.0>71.9	13.22	121/121	25/23	8/10
Diflubenzuron	311.1>158.1	311.1>141.0	16.19	61/61	19/47	10/8
Diflufenican	395.0>266.0	395.0>246.0	18.50	136/136	33/47	14/14
Dimefuron	339.0>166.9	339.0>256.0	13.96	141/141	29/23	10/14
Dimethachlor	256.0>224.0	256.0>148.1	13.07	46/46	19/35	12/8
Dimethenamide	276.0>244.0	276.0>168.0	14.16	51/51	19/33	14/10
Dimethoate	230.0>199.0	230.0>125.0	6.11	21/21	13/29	12/6
Dimethomorph	388.1>301.0	388.1>165.1	14.79/15.20	36/36	29/41	16/10
Dimoxystrobin	327.0>205.0	327.0>116.1	16.57	61/61	15/29	12/6
Diniconazole	326.1>70.1	326.1>158.9	17.70	25/25	63/39	8/10
Disulfoton	275.0>89.0	275.0>61.0	17.55	56/56	20/46	10/10
Disulfoton-sulfon	307.0>96.8	307.0>171.0	12.39	66/66	41/16	12/9
Diuron	233.0>72.0	233.0>46.1	11.42	86/86	23/39	8/12
EPN	324.1>295.9	324.1>157	18.14	81/81	19/31	16/10
Epoxiconazole	330.0>121.0	330.0>101.1	15.99	61/61	27/65	6/6
Etaconazole	328.1>159.0	328.1>123.0	15.76	61/61	37/75	10/6

Ethiofencarb	226.1>107.1	226.1>164.1	11.35	41/41	23/11	8/10
Ethiofencarb-sulfone	258.0>107.1	258.0>200.9	5.69	51/51	21/11	8/10
Ethiofencarb-sulfoxide	242.0>106.9	242.0>185.0	6.07	41/41	23/13	12/8
Ethiprole	397.0>350.8	397.0>254.9	14.54	116/116	29/47	18/14
Ethirimol	210.1>140.1	210.1>97.9	8.28	116/116	29/35	8/12
Ethofumesate	304.0>121.1	304.0>161.1	14.14	44/44	27/33	6/10
Ethoprophos	243.0>130.9	243.0>172.9	15.71	61/61	27/21	6/10
Etoxazole	360.1>141.0	360.1>304.0	20.38	100/100	35/25	6/16
Famoxadon	392.1>331.1	392.1>238.1	17.59	50/50	11/23	18/14
Fenamidone	312.1>92.0	312.1>65.0	14.57	56/56	39/71	8/10
Fenamiphos	304.1>217.0	304.1>202.0	16.42	101/101	31/47	12/12
Fenazaquin	307.1>161.1	307.1>147.0	21.04	71/71	23/27	10/7
Fenbuconazole	337.0>125.1	337.0>70.0	16.34	96/96	35/23	8/8
Fenchlorazol-ethyl	403.9>357.8	403.9>375.7	17.59	116/116	31/21	18/20
Fenfuram	202.0>109.0	202.0>120.0	10.82	91/91	27/21	8/6
Fenhexamid	302.1>97.1	302.1>55.0	15.59	86/86	31/57	6/8
Fenobucarb	208.1>95.1	208.1>152.1	13.75	36/36	21/11	8/10
Fenoxaprop-ethyl	362.0>288.0	362.0>121.0	18.78	116/116	25/37	16/15
Fenoxycarb	302.1>88.0	302.1>116.1	16.60	86/86	27/15	10/8
Fenpropidin	274.4>147.1	274.4>116.9	13.58	151/151	39/71	8/14
Fenpropimorph	304.2>147.1	304.2>117.1	13.95	91/91	39/77	8/8
Fenpyroximate	422.1>366.1	422.1>135.0	20.82	116/116	23/41	18/8
Fensulfothion-sulfon	325.0>268.9	325.0>296.9	13.47	101/101	21/15	14/18
Fenuron	165.1>72.0	165.1>46.1	5.67	91/91	21/19	10/8
Fipronil	454.0>367.9	454.0>254.8	16.58	60/60	31/51	18/14
Flazasulfuron	408.0>182.0	408.0>227.0	13.79	66/66	25/28	10/17
Flonicamid	230.0>173.9	230.0>147.9	3.40	81/81	25/37	10/8
Fluazifop	328.0>282.0	328.0>254.0	14.55	91/91	27/35	16/14
Flubendiamid	700.0>408.0	700.0>273.9	17.31	50/50	19/47	20/14
Fludioxonil	266.1>229.0	266.1>157.9	14.75	65/65	17/45	12/8
Flufenacet	364.0>194.1	364.0>152.0	15.84	56/56	15/25	10/8
Flumioxazin	355.0>327.2	355.0>299.0	13.86	136/136	20/38	18/16
Fluometuron	233.1>72.1	233.1>145.1	11.41	86/86	23/47	8/8
Fluopicolid	383.0>172.9	383.0>108.9	14.98	91/91	31/89	10/6
Fluoroglycofene-ethyl	465.0>343.8	465.0>222.9	18.77	51/51	19/45	18/12
Fluoxastrobin	459.0>427.0	459.0>188.0	16.08	101/101	25/45	22/10
Fluquinconazole	376.0>306.9	376.0>349.0	15.51	26/26	35/27	18/18
Fluridone	330.0>310.0	330.0>259.0	14.15	161/161	39/63	16/14
Flurochloridone	313.0>292.9	313.0>53.0	15.20	146/146	31/61	16/8
Fluroxypyr-meptyl	367.0>255.0	367.0>209.0	20.23	46/46	13/33	13/12
Flurtamone	334.1>247.0	334.1>178.1	14.54	136/136	37/59	14/10
Flusilazole	316.1>247.0	316.1>165.1	16.49	26/26	25/35	14/10

Fluthiacet-methyl	404.0>273.9	404.0>215.0	16.74	151/151	39/53	16/12
Flutolanil	324.1>262.0	324.1>242.0	15.07	16/16	25/35	14/14
Flutriafol	302.0>123.0	302.0>109.0	12.59	61/61	37/37	6/5
Forchlorfenuron	248.0>129.0	248.0>93.0	12.76	36/36	23/47	6/10
Formetanate	222.0>165.1	222.0>120.1	2.14	16/16	21/35	10/7
Fosthiazate	284.0>103.9	284.0>199.9	11.86	46/46	27/23	12/12
Fuberidazole	185.0>157.1	185.0>65.0	6.08	96/96	31/61	10/10
Furalaxyl	302.1>242.1	302.1>95.0	14.38	66/66	21/33	12/8
Furathiocarb	383.1>195.0	383.1>252.0	19.09	86/86	25/17	12/14
Halofenozide	331.0>105.0	331.0>275.0	14.50	60/60	23/15	6/12
Haloxyfop-ethoxyetyl	434.0>315.9	434.0>90.9	19.03	106/106	27/45	16/14
Haloxyfop-metyl	376.0>315.9	376.0>288.0	18.17	121/121	23/35	16/16
Hexaconazole	314.1>70.0	314.1>159.0	17.20	21/21	49/37	8/10
Hexaflumuron	461.0>158.1	461.0>141.0	18.84	111/111	23/63	10/8
Hexazinone	253.1>171.0	253.1>71.0	10.42	66/66	23/41	10/12
Hexythiazox	353.0>228.0	353.0>168.0	19.74	66/66	21/35	12/10
Imazalil	297.0>159.0	297.0>201.0	12.15	81/81	31/23	10/10
Imidacloprid	256.0>209.1	256.0>175.1	6.07	80/80	21/27	12/10
Indoxacarb	528.0>202.9	528.0>56.0	18.65	91/91	55/75	12/14
Iodosulfuron-methyl	507.9>167.0	507.9>141.0	13.89	71/71	25/40	10/8
Ipconazole	334.1>70.0	334.1>124.9	18.37	71/71	61/57	8/6
Iprovalicarb	321.2>119.1	321.2>203.2	15.58	61/61	23/12	8/10
Isocarbophos	307.0>231.0	307.0>272.9	13.04	50/50	21/9	12/14
Isoprocarb	194.1>95.0	194.1>137.0	12.07	36/36	21/13	16/8
Isoprothiolane	291.0>231.0	291.0>188.9	15.05	21/21	15/29	12/10
Isoproturon	207.1>72.0	207.1>46.1	12.44	91/91	23/37	10/10
Isoxaben	333.1>165.0	333.1>106.9	15.20	71/71	25/81	10/12
Isoxadifen-ethyl	313.2>232.1	313.2>204.2	16.69	34/34	27/39	12/12
Isoxaflutole	360.1>251.0	377.1>251.0	12.99	100/55	19/27	14/14
Kresoxim-methyl	314.1>206.0	314.1>116.1	16.67	70/70	10/19	10/8
Lenacil	235.1>153.0	235.1>136.0	12.45	66/66	21/45	8/8
Linuron	249.0>160.0	249.0>182.0	13.70	61/61	25/21	10/10
Lufenuron	511.0>158.1	511.0>140.9	20.03	111/111	25/73	10/8
Mandipropamid	412.1>328.0	412.1>356.0	15.30	96/96	21/15	18/18
Mefenacet	299.1>148.0	299.1>120.1	15.37	51/51	19/35	8/8
Mefenpyr-diethyl	373.1>326.9	373.1>160.1	17.37	70/70	23/47	16/10
Mepanipyrim	224.0>106.0	224.0>66.0	15.34	131/131	33/59	18/10
Mepronil	270.1>119.1	270.1>228.0	14.94	91/91	31/20	8/12
Mesosulfuron-methyl	504.0>182.0	504.0>83.0	13.08	101/101	29/81	10/10
Metalaxyl	280.1>220.1	280.1>160.1	12.90	41/41	19/31	12/10
Metamitron	203.1>175.1	203.1>104.0	5.88	96/96	23/31	10/12
Metazachlor	278.0>210.0	278.0>134.1	12.59	16/16	15/29	12/8

Metconazole	320.1>70.0	320.1>124.9	17.32	56/56	63/55	8/6
Methabenzthiazuron	222.1>165.0	222.1>150.0	12.12	46/46	23/43	10/8
Methacrifos	241.0>124.9	241.0>209.0	13.33	46/46	25/11	6/12
Methamidophos	142.0>94.0	142.0>124.9	0.63	36/36	21/19	6/6
Methfuroxam	230.0>137.0	230.0>111.1	13.32	81/81	27/21	8/8
Methiocarb	226.1>169.0	226.1>121.0	14.14	41/41	13/25	10/6
Methiocarb-sulfon	258.0>122.0	258.0>201.1	7.93	81/81	25/13	6/4
Methiocarb-sulfoxid	242.0>185.0	242.0>122.1	7.10	56/56	19/39	10/8
Methomyl	163.0>88.0	163.0>105.9	3.73	6/6	13/13	10/12
Methoprotryne	272.1>198.1	272.1>240.1	12.78	81/81	31/25	12/12
Methoxyfenozide	369.1>149.0	369.1>133.0	15.44	46/46	23/34	8/7
Metobromuron	259.0>169.9	259.0>148.0	11.68	66/66	25/21	10/8
Metolcarb	166.0>109.1	166.0>94.0	8.89	26/26	15/41	8/14
Metosulam	418.0>175.0	418.0>140.0	11.77	106/106	33/71	10/8
Metoxuron	229.0>72.0	229.0>46.1	8.83	76/76	21/37	8/8
Metrafenone	409.0>209.0	409.0>226.9	17.75	61/61	19/27	12/12
Metribuzin	215.1>187.1	215.1>84.1	9.49	96/96	25/27	10/10
Metsulfuron-methyl	382.0>167.0	382.0>198.9	11.28	61/61	21/29	10/12
Mevinphos	225.0>127.0	225.0>193.0	8.28	41/41	23/11	6/10
Mexacarbate	223.1>166.0	223.1>151.1	5.51	21/21	19/31	8/8
Monocrotophos	224.0>127.0	224.0>98.1	5.09	41/41	23/17	6/8
Monolinuron	215.0>125.9	215.0>99.0	10.98	61/61	23/45	6/12
Monuron	199.1>71.9	199.1>126.0	9.30	76/76	21/33	10/6
Myclobutanil	289.0>70.0	289.0>125.0	15.24	71/71	23/45	8/8
Neburon	275.0>88.1	275.0>114.1	16.44	101/101	21/21	10/8
Nicosulfuron	411.1>182.1	411.1>213.1	10.84	86/86	27/23	10/12
Nitenpyram	271.1>126.1	271.1>237.1	3.47	61/61	37/25	8/11
Norflurazon	304.1>283.9	304.0>160.0	13.10	131/131	33/43	16/10
Omethoate	214.0>124.9	214.0>182.9	1.90	46/46	29/15	6/10
Oxadixyl	279.1>219.1	279.1>133.1	9.81	46/46	15/29	12/8
OxamyI	237.0>72.0	237.0>90.0	3.68	25/25	31/11	8/8
Oxamyl-oxime	163.0>72.1	163.0>90.0	1.77	46/46	17/23	6/14
Oxycarboxin	268.0>174.9	268.0>147.0	7.28	66/66	31/21	10/10
Oxydemeton-methyl	247.0>169.0	247.0>124.9	4.31	41/41	19/29	10/6
Paclobutrazol	294.0>70.0	294.0>125.1	14.82	66/66	51/49	10/8
Penconazole	284.0>70.0	284.0>158.9	16.66	56/56	21/35	8/8
Pencycuron	329.0>124.9	331.0>126.9	17.89	70/70	29/31	6/6
Pendimethalin	282.0>212.0	282.0>194.0	19.63	16/16	15/25	12/10
Petoxamid	296.1>131.1	296.1>250.0	15.77	11/11	27/17	6/14
Phenmedipham	301.1>168.1	301.1>136.0	13.87	76/76	13/29	10/8
Phenthoate	321.0>79.0	321.0>163.0	16.71	46/46	57/16	10/8
Phorate-sulfon	293.0>171.0	293.0>96.8	12.28	51/51	15/47	8/14

Phorate-sulfoxid	277.0>198.9	277.0>142.9	12.02	31/31	13/27	12/8
Phosmet	318.0>160.0	318.0>133.0	13.73	46/46	17/51	10/6
Phoxim	299.0>77.0	299.0>129.0	17.58	46/46	43/15	12/6
Picoxystrobin	368.0>205.0	368.0>145.0	16.77	36/36	13/29	12/8
Piperonyl butoxide	356.2>177.0	356.2>119.1	19.35	26/26	17/47	10/8
Pirimicarb	239.1>72.0	239.1>182.1	8.66	56/56	37/21	10/10
Pirimicarb-desmethyl	225.1>72.0	225.1>168.0	6.05	56/56	33/19	12/10
Prochloraz	376.0>307.9	376.0>70.0	17.12	16/16	17/29	16/10
Promecarb	208.1>151.0	208.1>109.1	14.54	26/26	13/23	8/8
Prometon	226.1>142.0	226.1>184.1	11.21	81/81	31/25	8/10
Prometryn	242.1>158.0	242.1>200.1	13.97	76/76	31/25	10/12
Propamocarb	189.2>102.1	189.2>74.0	1.91	61/61	25/35	8/12
Propaquizafop	444.1>100.0	444.1>299.0	19.22	116/116	23/31	16/16
Propargite	368.1>231.1	368.1>175.1	20.31	41/41	13/21	12/10
Propham	180.1>138.0	180.1>91.9	11.79	50/50	13/31	8/10
Propiconazole	342.0>159.0	342.0>69.0	17.08	100/100	37/23	10/8
Propoxur	210.1>111.1	210.1>168.0	10.15	21/21	19/11	8/10
Propyzamide	256.0>189.9	256.0>172.9	14.41	66/66	19/31	12/10
Proquinazid	373.0>288.9	373.0>330.9	20.35	71/71	33/19	16/18
Prosulfocarb	252.1>91.0	252.1>128.1	18.43	66/66	35/17	10/6
Prosulfuron	420.0>141.0	420.0>167.0	14.66	76/76	25/25	8/10
Pyracarbolid	218.1>125.0	218.1>97.0	10.38	21/21	25/36	6/6
Pyraclostrobin	388.0>194.1	388.0>163.1	17.66	41/41	17/33	12/10
Pyrimethanil	200.1>107.0	200.1>82.0	12.21	96/96	33/32	12/9
Pyriproxyfen	322.0>96.1	322.0>185.0	19.52	61/61	21/31	8/10
Qinoxyfen	308.0>196.9	308.0>162.0	19.37	46/46	43/59	10/8
Quinalphos	299.0>163.0	299.0>147.0	16.55	66/66	31/31	10/8
Quinoclamine	208.0>104.9	208.0>76.9	9.57	96/96	33/53	16/10
Rimsulfuron	432.0>182.0	432.0>325.0	11.96	76/76	29/21	10/18
Rotenone	395.1>213.0	395.1>192.1	16.61	136/136	31/33	12/10
Secbumeton	226.1>170.1	226.1>100	11.2/11.45	81/81	25/39	10/12
Siduron	233.1>137.0	233.1>94.1	14.07/14.33	96/96	23/29	8/8
Simazine	202.1>132.0	202.1>104.0	10.13	61/61	25/33	8/12
Simetryn	214.1>124.1	214.1>144.0	10.23	86/86	27/28	8/7
Spinosyn A	732.4>142.1	732.4>98.0	18.01	136/136	35/103	8/16
Spinosyn D	746.4>142.1	746.4>98.1	18.70	146/146	35/101	8/16
Spirodiclofen	411.1>313.0	411.1>71.0	20.72	80/80	17/29	18/8
Spiromesifen	371.1>273.1	371.1>255.1	20.28	71/71	15/31	14/14
Spirotetramat	374.1>302.0	374.1>330.1	16.00	131/131	23/21	16/20
Spiroxamine	298.1>144.1	298.1>100.0	14.42	71/71	27/43	8/12
Sulfentrazone	387.0>306.9	387.0>145.9	11.31	146/146	29/55	16/8
Sulfometuron-methyl	365.0>150.0	365.0>107.0	11.23	66/66	23/59	8/12

Sulfosulfuron	471.0>211.0	471.0>261.0	14.11	76/76	19/23	12/12
Tebuconazol	308.1>70.0	308.1>125.1	16.83	41/41	57/59	8/8
Tebufenozide	353.1>133.1	353.1>297.1	16.74	41/41	25/11	6/16
Tebufenpyrad	334.1>117.1	334.1>145.0	19.18	121/121	45/35	8/9
Tebuthiuron	229.1>172.1	229.1>116.0	10.85	81/81	25/35	10/8
Teflubenzuron	381.0>158.0	381.0>141.0	19.18	81/81	21/49	10/8
Tepraloxydim	342.1>250.1	342.1>166.0	15.54	86/86	19/29	14/10
Terbumeton	226.0>170.0	226.0>114.1	11.19/11.44	81/81	25/33	10/8
Terbuthylazine	230.1>174.0	230.1>104.0	14.36	51/51	23/43	10/12
Terbutryne	242.1>186.1	242.1>91.0	14.19	76/76	25/35	10/10
Tetraconazole	372.0>159.0	372.0>70.0	16.00	26/26	37/73	10/10
Thiabendazol	202.0>175.0	202.0>131.1	5.50	121/121	37/45	10/8
Thiacloprid	253.0>126.0	253.0>72.9	8.22	96/96	29/81	6/8
Thiamethoxam	292.0>211.0	292.0>181.0	4.35	61/61	17/31	12/10
Thifensulfuron-methyl	388.0>167.0	388.0>204.9	10.89	61/61	21/35	8/12
Thiobencarb	258.1>125.0	258.1>89.0	17.53	36/36	25/69	6/10
Thiodicarb	355.0>88.0	355.0>108.0	12.70	51/51	27/21	10/8
Thiofanox-sulfone	268.1>75.9	251.1>75.9	6.42	50/50	15/15	10/10
Thiofanox-sulfoxide	252.1>104.0	252.1>57.0	6.09	50/50	16/35	9/8
Thiophanate-methyl	343.0>151.0	343.0>192.0	10.47	81/81	27/21	8/10
Tolclofos-methyl	301.0>268.9	301.0>174.9	17.47	61/61	23/35	14/10
Tolylfluanid	364.0>238.0	364.0>137.0	16.91	100/100	19/39	12/8
Tralkoxydim	330.1>284.1	330.1>138	19.67	76/76	17/27	14/6
Triadimefon	294.1>197.1	294.1>225	15.09	51/51	21/17	12/11
Triadimenol	296.0>70.0	296.0>227.0	15.34	56/56	19/15	8/8
Triasulfuron	402.0>167.1	402.0>141.0	11.02	81/81	23/27	10/8
Triazoxide	248.0>95.0	248.0>68.0	10.57	141/141	36/35	7/8
Tribenuron-methyl	396.0>155.0	396.0>181.0	13.05	76/76	19/27	8/10
Tricyclazole	190.0>163.0	190.0>136.0	8.62	96/96	31/39	10/8
Trifloxystrobin	409.1>186.0	409.1>206.1	18.57	61/61	25/19	10/12
Triflumizole	346.0>278.0	346.0>73.0	18.26	25/25	15/21	18/10
Triflumuron	359.0>156.0	359.0>139.0	17.67	56/56	21/45	10/8
Triflusulfuron-methyl	493.0>264.0	493.0>238.0	15.32	76/76	29/35	14/12
Triforine	434.9>389.8	434.9>98.0	13.89/14.22	60/60	17/43	20/14
Triticonazole	318.0>70.0	318.0>125.0	15.71	71/71	49/47	8/8
Uniconazole	292.1>70.0	292.1>125.0	16.18	106/106	59/37	10/8
Vamidothion	288.0>146.0	288.0>117.9	7.10	36/36	17/33	8/9
Zoxamide	336.1>186.9	336.1>159	16.90	46/46	31/55	10/10
Carbendazim-D4	196.1>164.1	196.1>136.1	4.10	66/66	25/41	10/8
Atrazine-D5	221.1>179.1	221.1>69.0	12.08	101/101	25/51	10/10
Linuron-D6	255.0>160.0	255.0>185.0	13.64	81/81	25/21	10/10

[1] Retention time (RT); [2] Declustering potential (DP); [3] Collision energy (CE); [4] Cell exit potential (CXP).

Table S2. Retention times (RT) and detector type of each pesticide analysed by GC-ECD/NPD.

**GC system**	**GC Agilent 6890N with electron capture and nitrogen–phosphorus detectors**	
**Injection Mode**	**Splitless**	
**Target compound**	**Detector**	**RT[1] (min)**
Acetochlor	ECD/NPD	14.741
Acrinathrin	ECD/NPD	20.853/21.151
Aldrin	ECD	15.789
Azinphos-ethyl	ECD/NPD	21.656
Azinphos-methyl	ECD/NPD	20.813
Bifenthrin	ECD	19.781
Bromophos-ethyl	ECD/NPD	16.899
Bromophos-methyl	ECD/NPD	16.114
Bromopropylate	ECD	19.913
Captan	ECD	16.621
Chlorfenvinphos	ECD/NPD	16.463
Chlorothalonil	ECD	14.204
Chlorpropham	NPD	12.174
Chlorpyrifos	ECD/NPD	15.666
Chlorpyrifos-methyl	ECD/NPD	14.820
Chlozolinate	ECD/NPD	16.387
Cyanazine	ECD/NPD	15.663
Cyfluthrin	ECD	23.294/23.461/23.700
Beta-Cyfluthrin	ECD	23.289/23.464/23.705
Lambda-Cyhalothrin	ECD	20.742/21.011
Alpha-Cypermethrin	ECD	23.983/24.380
Cypermethrin	ECD	23.981/24.181/24.417
Zeta-Cypermethrin	ECD	23.984/24.251/24.395
DDD-p,p'	ECD	18.310
DDE-p,p'	ECD	17.535
DDT-o,p'	ECD	18.388
DDT-p,p'	ECD	18.986
Deltamethrin	ECD	28.031/28.765
Diazinon	ECD/NPD	13.754
Dichloran	ECD	13.194
Dicofol	ECD	15.865
Dieldrin	ECD	17.703
Alpha-Endosulfan	ECD	17.223
Beta-Endosulfan	ECD	18.272
Endosulfan-sulfate	ECD	19.021
Endrin	ECD	18.119

Esfenvalerate	ECD	26.357/26.955
Ethion	ECD/NPD	18.291
Fenarimol	ECD/NPD	21.452
Fenchlorphos	ECD/NPD	15.110
Fenitrothion	ECD/NPD	15.320
Fenpropathrin	ECD/NPD	19.977
Fenthion	NPD	15.637
Fenvalerate	ECD	26.419/27.018
Tau-Fluvalinate	ECD	26.942/27.020
Folpet	ECD	16.755
Formothion	ECD/NPD	14.367
Alpha-HCH	ECD	12.966
Beta-HCH	ECD	13.538
Gamma-HCH (Lindane)	ECD	13.640
Heptachlor	ECD	15.092
Heptachlor endo-epoxide	ECD	16.602
Heptachlor exo-epoxide	ECD	16.523
Heptenophos	NPD	11.359
Hexachlorobenzene (HCB)	ECD	13.169
Imibenconazole	ECD	32.431
Iprodione	ECD/NPD	18.167
Isofenphos	ECD/NPD	16.462
Isofenphos-methyl	ECD/NPD	16.195
Malaoxon	ECD/NPD	14.677
Malathion	ECD/NPD	15.434
Mecarbam	ECD/NPD	16.454
Methidathion	ECD/NPD	16.867
Methoxychlor	ECD	20.005
Metolachlor	ECD/NPD	15.671
Napropamide	NPD	17.247
Nitrofen	ECD	17.953
Parathion	ECD/NPD	15.745
Parathion-methyl	ECD/NPD	14.820
Permethrin	ECD	22.239/22.450
Phosalone	ECD/NPD	20.684
Pirimiphos-ethyl	NPD	15.991
Pirimiphos-methyl	NPD	15.229
Procyazine	ECD/NPD	16.857
Procymidone	ECD/NPD	16.646
Profenofos	ECD/NPD	17.417
Propachlor	ECD/NPD	11.865
Propazine	ECD/NPD	13.354

Pyrazophos	ECD/NPD	21.436
Pyridaben	ECD/NPD	22.587
Quintozene	ECD	13.741
Tefluthrin	ECD	13.921
Tetrachlorvinphos	ECD/NPD	17.996
Tetradifon	ECD	20.559
Tetramethrin	ECD	19.682/19.801
Triazophos	NPD	18.470
Trichlorfon	ECD/NPD	7.354
Trifluralin	ECD/NPD	12.367
Vinclozolin	ECD/NPD	14.789

[1] Retention time (RT).

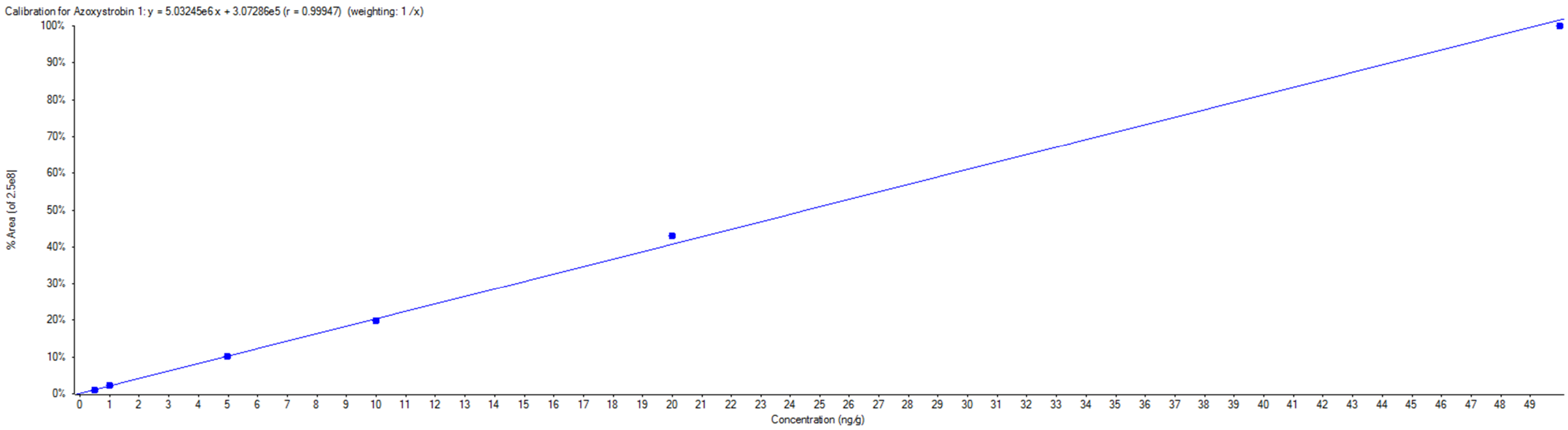

(**a**)

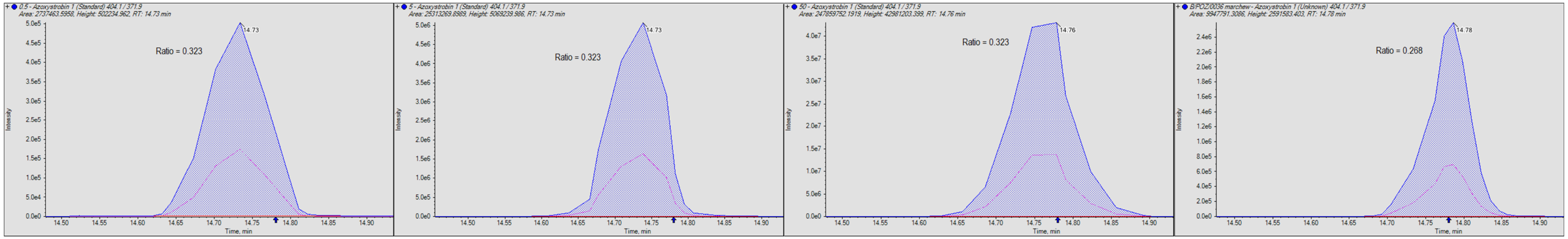

(**b**) (**c**) (**d**) (**e**)

Figure S1. Calibration curve and extracted ion chromatograms of azoxystrobin (MultiQuant software): (**a**) Calibration curve (0.5–50 ng/mL); (**b**) Extracted ion chromatogram at 0.5 ng/mL; (**c**) Extracted ion chromatogram at 5 ng/mL; (**d**) Extracted ion chromatogram at 50 ng/mL; (**e**) Extracted ion chromatogram of azoxystrobin residues at 0.019 mg/kg (the carrot sample).

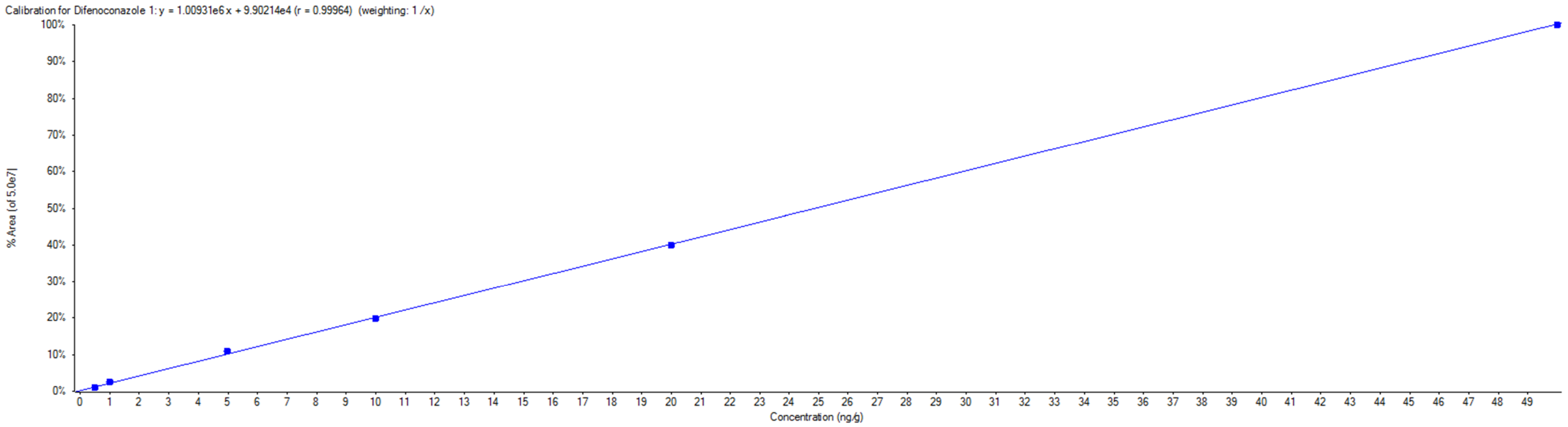

(**a**)

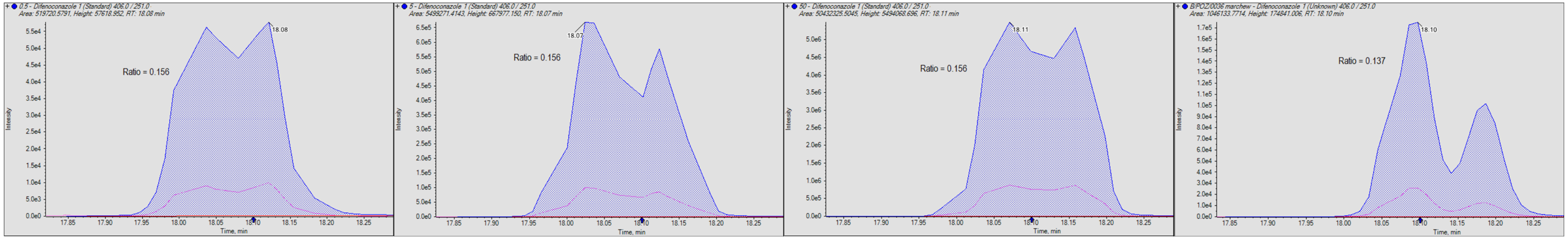

(**b**) (**c**) (**d**) (**e**)

Figure S2. Calibration curve and extracted ion chromatograms of difenoconazole (MultiQuant software): (**a**) Calibration curve (0.5–50 ng/mL); (**b**) Extracted ion chromatogram at 0.5 ng/mL; (**c**) Extracted ion chromatogram at 5 ng/mL; (**d**) Extracted ion chromatogram at 50 ng/mL; (**e**) Extracted ion chromatogram of difenoconazole residues at 0.010 mg/kg (the carrot sample).

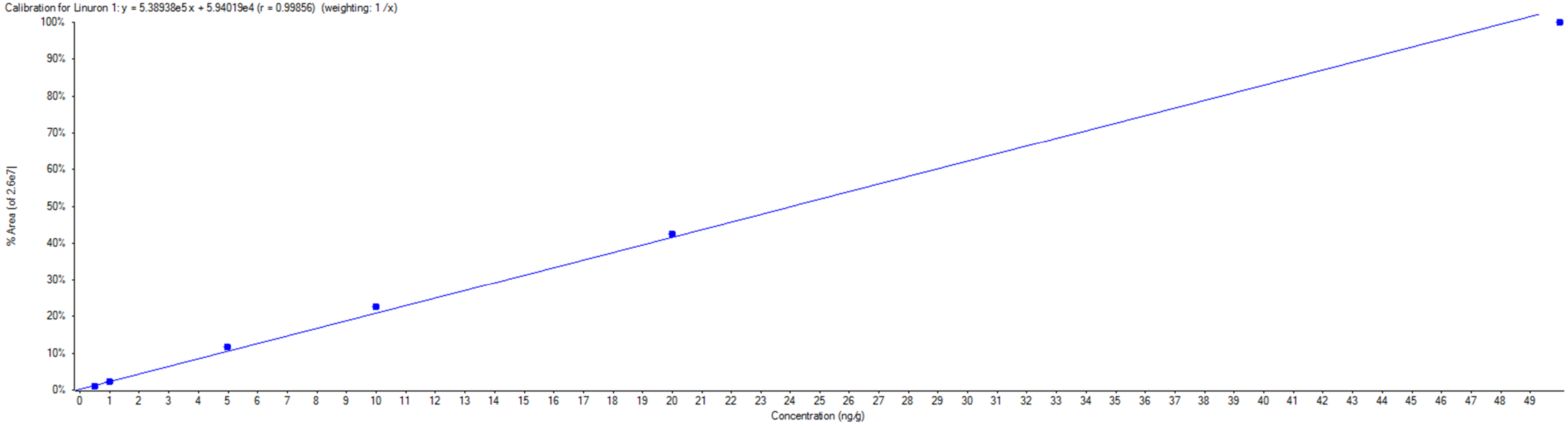

(**a**)

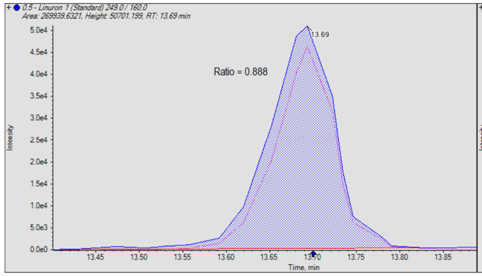

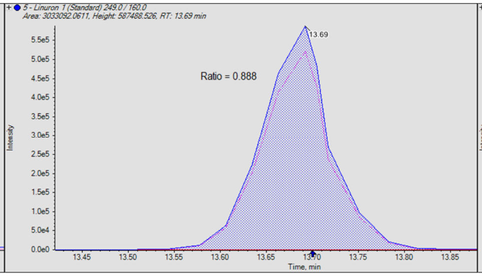

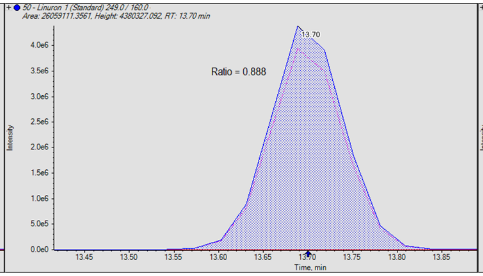

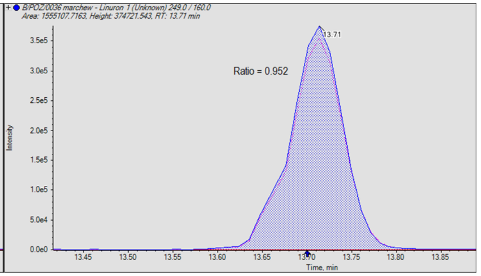

(**b**) (**c**) (**d**) (**e**)

Figure S3. Calibration curve and extracted ion chromatograms of linuron (MultiQuant software): (**a**) Calibration curve (0.5–50 ng/mL); (**b**) Extracted ion chromatogram at 0.5 ng/mL; (**c**) Extracted ion chromatogram at 5 ng/mL; (**d**) Extracted ion chromatogram at 50 ng/mL; (**e**) Extracted ion chromatogram of linuron residues at 0.027 mg/kg (the carrot sample).
